# Fluence and Temperature Dependences of Laser-Induced Ultrafast Demagnetization and Recovery Dynamics in *L*1_0_-FePt Thin Film

**DOI:** 10.3390/ma16145086

**Published:** 2023-07-19

**Authors:** Zhikun Xie, Yuanhai Cai, Meng Tang, Jielin Zhou, Junhao Liu, Jun Peng, Tianran Jiang, Zhong Shi, Zhifeng Chen

**Affiliations:** 1 School of Physics and Materials Science, Guangzhou University, Guangzhou 510006, China; 2 State-Key Laboratory of Optoelectronic Materials and Technologies, School of Physics, Sun Yat-Sen University, Guangzhou 510275, China; 3 Shanghai Key Laboratory of Special Artificial Microstructure Materials and Technology, School of Physics Science and Engineering, Tongji University, Shanghai 200092, China

**Keywords:** magnetization dynamics, alloy films, ultrafast demagnetization, temperature dependence, magneto-optical Kerr effect

## Abstract

The fundamental mechanisms of ultrafast demagnetization and magnetization recovery processes in ferromagnetic materials remain incompletely understood. The investigation of different dynamic features which depend on various physical quantities requires a more systematic approach. Here, the femtosecond laser-induced demagnetization and recovery dynamics in *L*1_0_-Fe_0.5_Pt_0.5_ alloy film are studied by utilizing time-resolved magneto-optical Kerr measurements, focusing on their dependences of excitation fluence and ambient temperature over broad ranges. Ultrafast demagnetization dominated by Elliott-Yafet spin-flip scattering, and two-step magnetization recovery processes are found to be involved in all observations. The fast recovery time corresponding to spin–lattice relaxation is much shorter than that of many ferromagnets and increase with excitation fluence. These can be ascribed to the strong spin–orbit coupling (SOC) demonstrated in FePt and the reduction of transient magnetic anisotropy, respectively. Surprisingly, the demagnetization time exhibits no discernible correlation with ambient temperature. Two competitive factors are proposed to account for this phenomenon. On the other hand, the spin–lattice relaxation accelerates as temperature decreases due to enhanced SOC at lower ambient temperature. A semiquantitative analysis is given to get a visualized understanding. These results offer a comprehensive understanding of the dynamic characteristics of ultrafast demagnetization and recovery processes in iron-based materials with strong SOC, highlighting the potential for regulating the magnetization recovery process through temperature and laser fluence adjustments.

## 1. Introduction

Ever since the discovery of ultrafast demagnetization phenomenon excited by femtosecond laser pulses in nickel thin film [1], the study of magneto-optical interactions has become an exciting frontier area due to its scientific significance and potential applications in high-speed magnetic and spintronic devices technology [2]. Specifically, the dynamic characteristics of laser-induced ultrafast demagnetization and magnetization recovery processes in diverse material systems and the underlying physical mechanisms have attracted much attention [3,4,5,6,7,8,9,10,11,12,13,14]. However, despite numerous experimental and theoretical studies [15,16,17,18,19,20,21,22], there are still ongoing debates surrounding the mechanism of ultrafast demagnetization.

Time-resolved magneto-optical Kerr (TR-MOKE) technology has been proven to be a powerful tool for investigating magnetization dynamics. A prominent study by Roth et al. using this technology demonstrated that increasing the excitation light intensity or elevating the ambient temperature can induce a transition from one-step to two-step demagnetization process in nickel, which is consistent with the expectation based on the Elliott-Yafet (EY) type electron–phonon scattering mechanism [23]. By combining photoemission spectroscopies and TR-MOKE measurements across a wide range of excitation fluence, You et al. revealed the mechanism behind the ultrafast magnetic phase transition [24]. Atxitia theoretically demonstrated that a high magnetic field combined with elevated temperature can speed up the ultrafast magnetization dynamics [25]. These studies indicate that modifications in experimental parameters, such as ambient temperature, excitation fluence, and external magnetic field, can lead to distinct magnetization dynamics even within the same material system, and thus help to provide further insight into the relative dynamic mechanisms. However, there is a lack of relevant comprehensive research, particularly with regards to the temperature-dependent demagnetization and recovery dynamics in various materials.

FePt with a *L*1_0_ phase exhibits exceptionally strong magnetocrystalline anisotropy and high Curie temperature, rendering it an optimal material for ultrahigh-density thermal-assisted magnetic recording [26,27]. Therefore, investigation on its ultrafast magnetization dynamics is of great significance. Additionally, the strong spin–orbit coupling (SOC) in this material [28,29] can facilitate the exploration of the impact of strong SOC on ultrafast demagnetization and magnetization recovery processes.

In the present work, we study the femtosecond laser-induced demagnetization and recovery dynamics in *L*1_0_-Fe_0.5_Pt_0.5_ alloy film utilizing TR-MOKE measurements, varying the excitation fluence and ambient temperature over broad ranges. We demonstrate that the ultrafast demagnetization process is dominated by E-Y scattering, and two-step magnetization recovery processes are involved in all observations. The spin–lattice relaxation time is found to be much shorter than that of many ferromagnets and increases with excitation fluence, whereas the demagnetization time does not show any discernible correlation with ambient temperature. We present explanations for these phenomena and offer a semiquantitative analysis to facilitate comprehension. Our findings offer a comprehensive understanding of the dynamic characteristics of ultrafast demagnetization and recovery processes in iron-based materials with strong SOC.

## 2. Materials and Experimental Methods

### 2.1. Sample Preparation and Characterization

**Figure 1:**
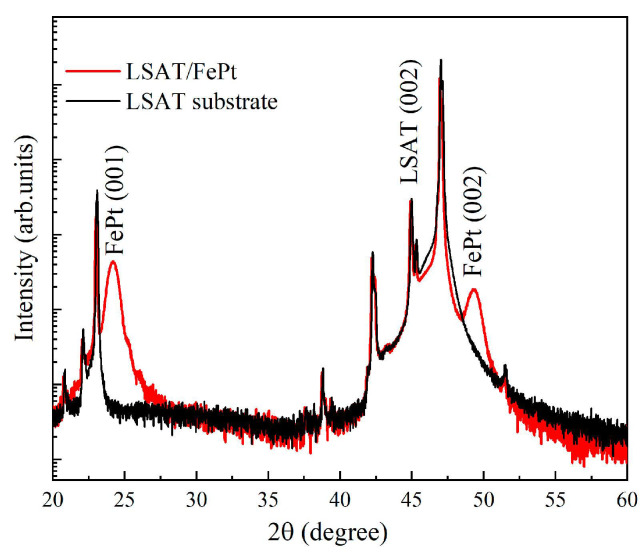
XRD patterns of the LSAT/FePt sample and the bare LSAT substrate.

The sample is a single-layer *L*1_0_-Fe_0.5_Pt_0.5_ film with thickness of 11.9 nm. The film was epitaxially grown on a 5 × 5 mm^2^ (La,Sr)(Al,Ta)O_3_ (LSAT) substrate using magnetron sputtering in a uniform DC field at a temperature of 400 °C. After the deposition process, the film was annealed at a temperature of 750 °C under vacuum conditions (2.7 × 10^−8^ Torr). The presence of diffraction peaks of FePt (001) and (002) at 2θ = 24.3° and 2θ = 49.4°, respectively, confirm the formation of a face-centered tetragonal (fct) *L*1_0_-FePt phase [30,31,32], while the extra peaks correspond to LAST substrate as shown in Figure 1.

### 2.2. TR-MOKE Measurement

**Figure 2:**
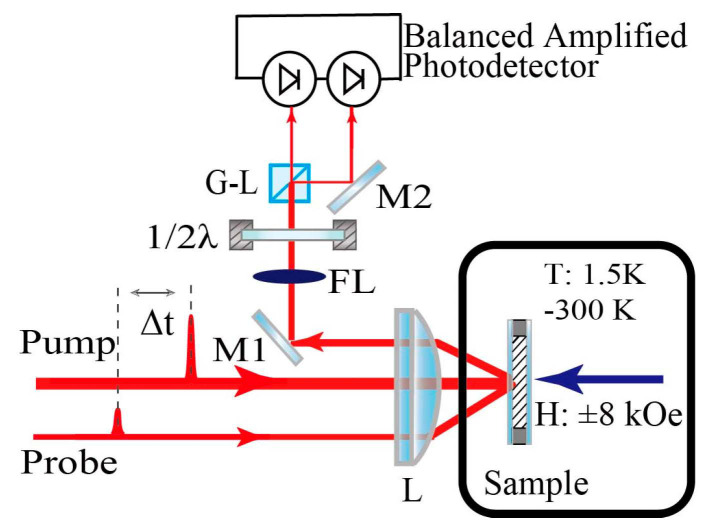
Simplified schematic of the TR-MOKE measurement setup. L: lens. M1 and M2: mirrors. FL: neutral-density filter. 1/2λ: half-wave plate. G-L: Glan prism. ∆t: time delay between the pump and probe pulses controlled by a motorized translation stage on the pump optical path.

The TR-MOKE setup depicted in Figure 2 was employed to investigate the ultrafast magnetization dynamics. The linearly polarized laser pulses with a central wavelength of 800 nm and a duration of 60 fs were generated at a repetition rate of 1 kHz by a Ti:sapphire amplifier. The laser beam was split into pump and probe pulses with the ratio of pump-to-probe at power of 40. The sample was placed in a superconductivity magnet. The temperature of the sample could be adjusted within a range of 1.5–300 K, and the external magnetic field was applied within a range of ±8kOe. The pump pulses were normally incident and focused to a spot of ~150 μm diameter on the sample surface, while the probe pulses were incident at a small angle and focused to a spot of ~75 μm diameter on the sample surface. The polar Kerr rotation of the probe pulses reflected from the sample surface was measured by a balanced optical bridge combined with a lock-in amplifier. The transient Kerr rotation, Δ*θ*(*t*), is defined as the difference between Kerr rotation angles of the probe reflected from sample after and before pumping the sample, i.e., Δ*θ*(*t*) = *θ*(*t*) − *θ*_0_, where *θ*(*t*) denotes the Kerr rotation at a delay time *t*, while *θ*_0_ is the initial Kerr rotation before pumping. Δ*θ*(*t*) is approximatively proportional to the magnetization change at *t*.

## 3. Results and Discussion

### 3.1. Hysteresis Loops and Coercivity

**Figure 3:**
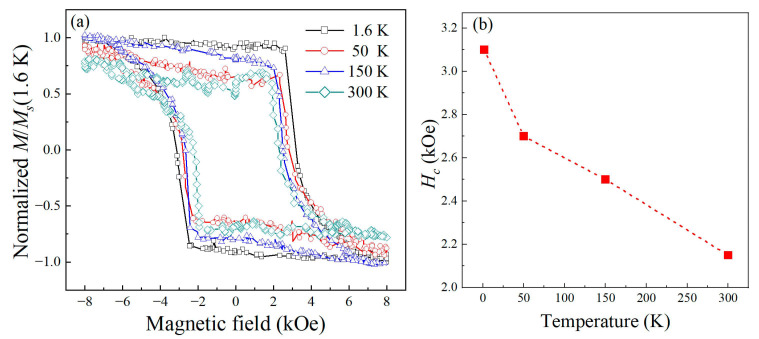
(**a**) Out-of-plane normalized Kerr hysteresis loops of the FePt film at different ambient temperatures. (**b**) Coercivity versus ambient temperature.

The Kerr hysteresis loops of *L*1_0_-FePt alloy film were first measured under a variable magnetic field applied perpendicularly to the film plane and plotted in Figure 3a. The normalized out-of-plane hysteresis loops present nearly square and centric-symmetric shapes at all temperatures, which reveal that the sample has a strong perpendicular magnetic anisotropy. In addition, the coercivity of the films decreases from ~3.12 kOe at 1.6 K to ~2.15 kOe at 300 K as shown in Figure 3b, indicating an enhancement in magnetic anisotropy with the decrease in the ambient temperature [33,34]. According to the Heisenberg model [28,35], magnetic anisotropy is originated from SOC. Therefore, the temperature dependence of hysteresis loops indicates that the SOC strength of *L*1_0_-FePt film increases with decreasing ambient temperature.

### 3.2. Fluence-Dependent Dynamics

**Figure 4:**
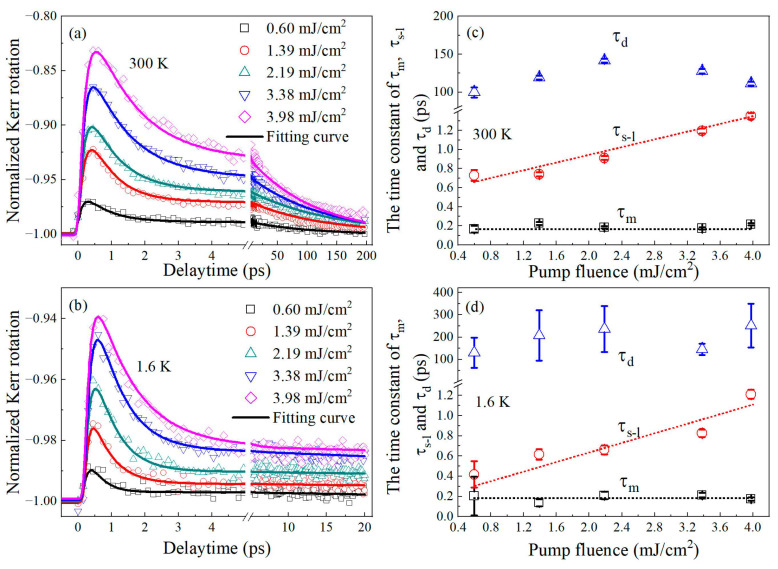
(**a**,**b**) Fluence-dependent demagnetization and recovery dynamics of the FePt film measured at two different ambient temperatures. The solid lines represent the best fittings of the dynamics. (**c**,**d**) Different characteristic time constants extracted from the dynamics in (**a**,**b**), respectively.

We measured the laser-induced demagnetization and magnetization recovery dynamics under a saturation field of 8 kOe and different pump fluences. Figure 4a,b show the fluence-dependent magnetization dynamics of FePt at 1.6 K and 300 K, respectively. The temporal traces of FePt at both ambient temperatures exhibit ultrafast demagnetization, followed by two magnetization recovery processes as the excitation fluence varies from 0.60 to 3.98 mJ/cm^2^. The ultrafast demagnetization of 3d ferromagnetic materials was generally attributed to the E-Y spin-flip scattering [8,23]. The rapid magnetization recovery is likely a result of spin–lattice relaxation [1,5], and the slow magnetization recovery attributed to the heat diffusion from the sample into the substrate and surroundings [36].

To understand the observed dynamic behaviors, it is necessary to extract the time constants of the magnetization processes. A phenomenological model including demagnetization processes and two magnetization recovery processes is proposed here, and written as:

(1) $$F=C(t)⨂\left\{\varepsilon (t)\cdot A_{m}\left[1-\exp\left(-\frac{t}{\tau_{m}}\right)\right]\cdot \left[A_{s-l}\exp\left(-\frac{t}{\tau_{s-l}}\right)+\left(1-A_{s-l}\right)\cdot \exp\left(-\frac{t}{\tau_{d}}\right)\right]\right\}$$

where the first square bracket term describes the dynamics of ultrafast demagnetization with an amplitude *A_m_* and a time constant *τ_m_*. The second square bracket term describes the dynamics of fast and slow magnetization recovery with a fast recovery amplitude *A_s−l_* and two time-constants *τ_s−l_* and *τ_d_*. The function *ε*(*t*) represents a step function, while *C*(*t*) stands for the cross-correlation function of pump and probe pulses, approximately assumed to be a Gaussian function. The symbol ⨂ denotes the convolution operation.

The magnetization dynamics of FePt alloys at 1.6 K and 300 K can be fitted well with Equation (1), as colored solid lines shown in Figure 4a and 4b, respectively. The three extracted parameters, *τ_m_*, *τ_s−l_*, and *τ_d_* as a function of pump fluence are plotted in Figure 4c,d by the scattered points. Compared to Figure 4a,b, the fluence dependence trends of the three time-constants do not show significant changes with temperature. The ultrafast demagnetization processes at both temperatures present time constants (*τ_m_*) of approximately 200 fs, without obvious dependence of pump fluence, differing from what would be expected via the electron–phonon-mediated spin-flip scattering [8,23,29]. It should be noted that the increased demagnetization rates by increasing pump fluences here were maintained within a small variation range, which was insufficient to cause a significant decrease in spin-flip probability. Specifically, the demagnetization rates range only from 0.03 to 0.17 at 300 K and from 0.01 to 0.06 at 1.6 K.

The value of *τ_s−l_* for *L*1_0_-FePt film here was determined to fall within 2 ps as shown in Figure 4c,d, being much shorter than that for lots of ferromagnets. We can attribute this result to the large SOC strength in *L*1_0_-Fe_0.5_Pt_0.5_, because stronger spin–orbit interaction evidently leads to faster spin–lattice relaxation [29]. Additionally, at both 1.6 K and 300 K, *τ_s−l_* increases with increasing excitation fluence, ranging within 0.4–1.2 ps and 0.7–1.4 ps, respectively. *τ_s−l_* is related to the magnetic anisotropy as:

(2) $$\tau_{s-l}=1/\left[A_{\theta_{D}}{\left|E_{a}\right|}^{2}\right]$$

where $A_{\theta_{D}}$ is a factor reflecting the accessible phonon population and $E_{a}$ is the magnetic anisotropy energy [34]. The increase of pump fluence increases the agitations of spins, leading to more reduction of the transient $E_{a}$. Since this process should be much faster than the phonon generation, the transient change of $A_{\theta_{D}}$ can be neglected for this time interval. In other words, the slower spin–lattice relaxation observed at higher excitation fluence is mainly attributed to the weakening of transient magnetic anisotropy.

Moreover, it is noteworthy that the *τ_s−l_* at 300 K is longer than that at 1.6 K for all excitation fluences, suggesting that the larger SOC strength at lower temperature accelerates the spin–lattice relaxation in FePt film. We will further analyze this important results in the following parts.

We did not observe significant pump fluence dependence on the time constant of slow magnetization recovery(*τ_d_*) at both 1.6 K and 300 K. This is reasonable because the pump fluence used in our experiment is not strong enough to raise the equilibrium temperature of the sample and substrate significantly. Therefore, the transfer of thermal energy between the sample and the substrate basically remains unchanged in our experiment.

### 3.3. Temperature-Dependent Dynamics

**Figure 5:**
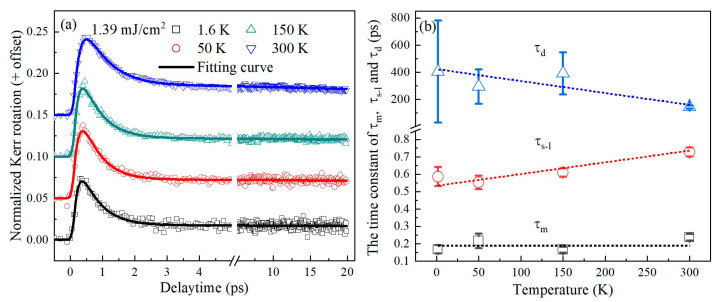
(**a**) Temperature-dependent demagnetization and recovery dynamics of FePt alloys measured in 1.39 mJ/cm^2^. (**b**) Different characteristic time constants extracted from the dynamics in (**a**) versus the ambient temperature.

To further confirm the above finding about demagnetization and magnetization recovery at the two different temperatures, we studied the magnetization dynamics of FePt alloys with the same excitation fluence and different ambient temperature ranging from 1.6 K to 300 K, as shown in Figure 5a. All dynamic traces exhibit an ultrafast demagnetization process, as well as fast and slow magnetization recovery processes as mentioned above. It is worth noting that the maximum amplitude of demagnetization slightly increases from 0.069 to 0.094 as the ambient temperature rises, being consistent with previous reports [23]. We globally fit the temperature-dependent dynamics using Equation (1), as shown by the solid lines in Figure 5a. *τ_m_*, *τ_s-l_*, and *τ_d_* are extracted and shown in Figure 5b.

The value of *τ_m_* is surprisingly found to remain almost unchanged around ~200 fs. This can be explained by the combined influence of two factors i.e., electron–phonon scattering and SOC, in the temperature-dependent dynamics of ultrafast demagnetization. The increase in electron–phonon scattering with rising ambient temperature leads to a longer demagnetization time, whereas the weaker SOC of FePt alloys at higher temperatures leads to faster demagnetization. These two factors counteract each other, resulting in no noticeable temperature dependence of *τ_m_*.

On the other hand, *τ_d_* exhibits a considerable dependence on temperature (decreasing from 405 to 143 ps), which is expected. The duration of thermal transports from FePt to the substrate can be analyzed based on the fact that *τ_d_* ∝ 1/*κ* [37], where FePt’s thermal conductivity (*κ*) increases as temperature increases [38]. As a result, the transfer of thermal energy from the sample to the substrate is accelerated with increasing temperature.

As shown in Figure 5b, *τ_s−l_* exhibits a decreasing trend as the ambient temperature decreases. As discussed previously, the sample at lower temperature demonstrated stronger SOC. It is reasonable to infer that stronger SOC would accelerate the spin–lattice relaxation. Further analysis can be carried out by considering the magnetic anisotropy, which is positively correlated with the strength of the SOC. The Kerr loops shown in Figure 2 reveal that the perpendicular magnetic anisotropy increases with decreasing ambient temperature. Assuming that factors such as defects and dislocations, which would influence the pinning sites in domain wall motion, can be neglected, the coercivity $H_{c}$ and the magnetic anisotropy energy $E_{a}$ have an approximate simple relation: $H_{c}\propto E_{a}$ [39], which is consistent with the report by the ferromagnetic resonance [40]. By combining this with the relationship given by Equation (2), one can obtain a relation of $\tau_{s-l}\propto {\left({A_{\theta_{D}}\cdot H}_{c}\right)}^{-2}$. Note that since $A_{\theta_{D}}$ keeps minor variation in a wide temperature range, one can approximatively regard $A_{\theta_{D}}$ as a constant.

**Figure 6:**
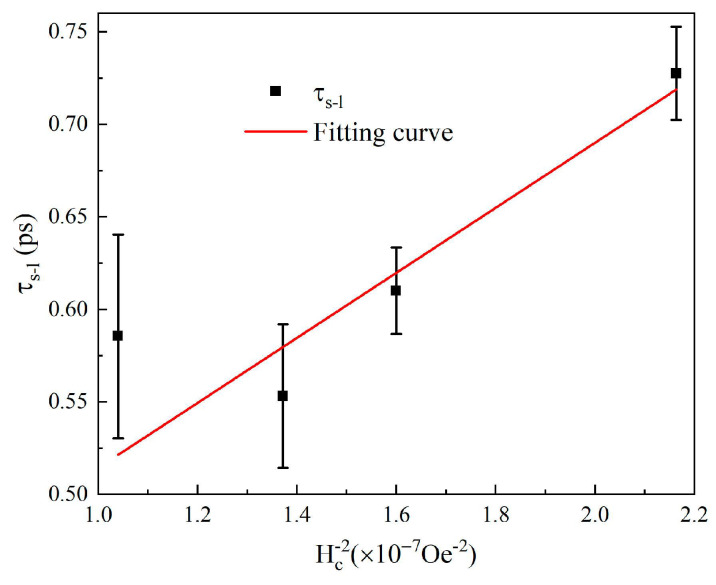
Coercivity dependence of *τ_s−l_*. The solid line represents the fitting by the analytical model.

The coercivity dependence of *τ_s−l_* can be fitted basically by this model, as the red solid line shown in Figure 6, further supporting the conclusion that stronger SOC at lower temperatures is responsible for the accelerated spin–lattice relaxation. However, a significant fitting deviation occurs at ${H_{c}}^{-2}=1.04\times 10^{-7}{Oe}^{-2}$ corresponding to an ambient temperature of 1.6 K. This may be mainly attributed to two factors. Firstly, the factor $A_{\theta_{D}}$ will decrease sharply at extremely low ambient temperature due to phonon freezing, resulting in the spin–lattice process slowing down. Secondly, the microscopic model of Equation (2) may be not suitable at temperatures far below the Debye temperature [34]. Despite this deviation, this semi-quantitative analysis provides a way to obtain a visualized understanding of the modulation of the first-step magnetization recovery by magnetic anisotropy or SOC strength, with varying ambient temperature.

## 4. Conclusions

We conducted a study on the femtosecond laser-induced demagnetization and recovery dynamics in an *L*1_0_-Fe_0.5_Pt_0.5_ alloy film through a time-resolved magneto-optical Kerr measurement. We focused on the influence of excitation fluence and ambient temperature on the demagnetization and recovery dynamics. Our findings demonstrate that the Elliott-Yafet spin-flip scattering dominates the ultrafast demagnetization process, and two-step magnetization recovery processes are involved in all observations. The spin–lattice relaxation time ranging within 2 ps is much shorter than that of many ferromagnets due to the strong SOC in the material. Surprisingly, we found that the demagnetization time was not affected by ambient temperature. This can be explained by two competitive factors, i.e., E-Y spin-flip scattering and SOC. In contrast, the spin–lattice relaxation accelerates as temperature decrease due to the enhanced SOC. A semiquantitative analysis was performed to provide a visual understanding. Our findings contribute to a comprehensive insight into the dynamic characteristics of ultrafast demagnetization and recovery in iron-based materials with strong SOC, and suggest the potential for regulating these processes through temperature adjustments.

## Data Availability

The data presented in this study are available on reasonable request from the corresponding author.
